# Supplementary material for: Interpreting mammalian synonymous site conservation in light of the unwanted transcript hypothesis
Source: Nat Commun. 2025 Feb 27;16:2007. doi: 10.1038/s41467-025-57179-w (PMC11865589; doi:10.1038/s41467-025-57179-w)
Supplement: Supplementary file 1 — Supplementary Information [file 41467_2025_57179_MOESM1_ESM.pdf]

Supplementary information

Supplementary Figures

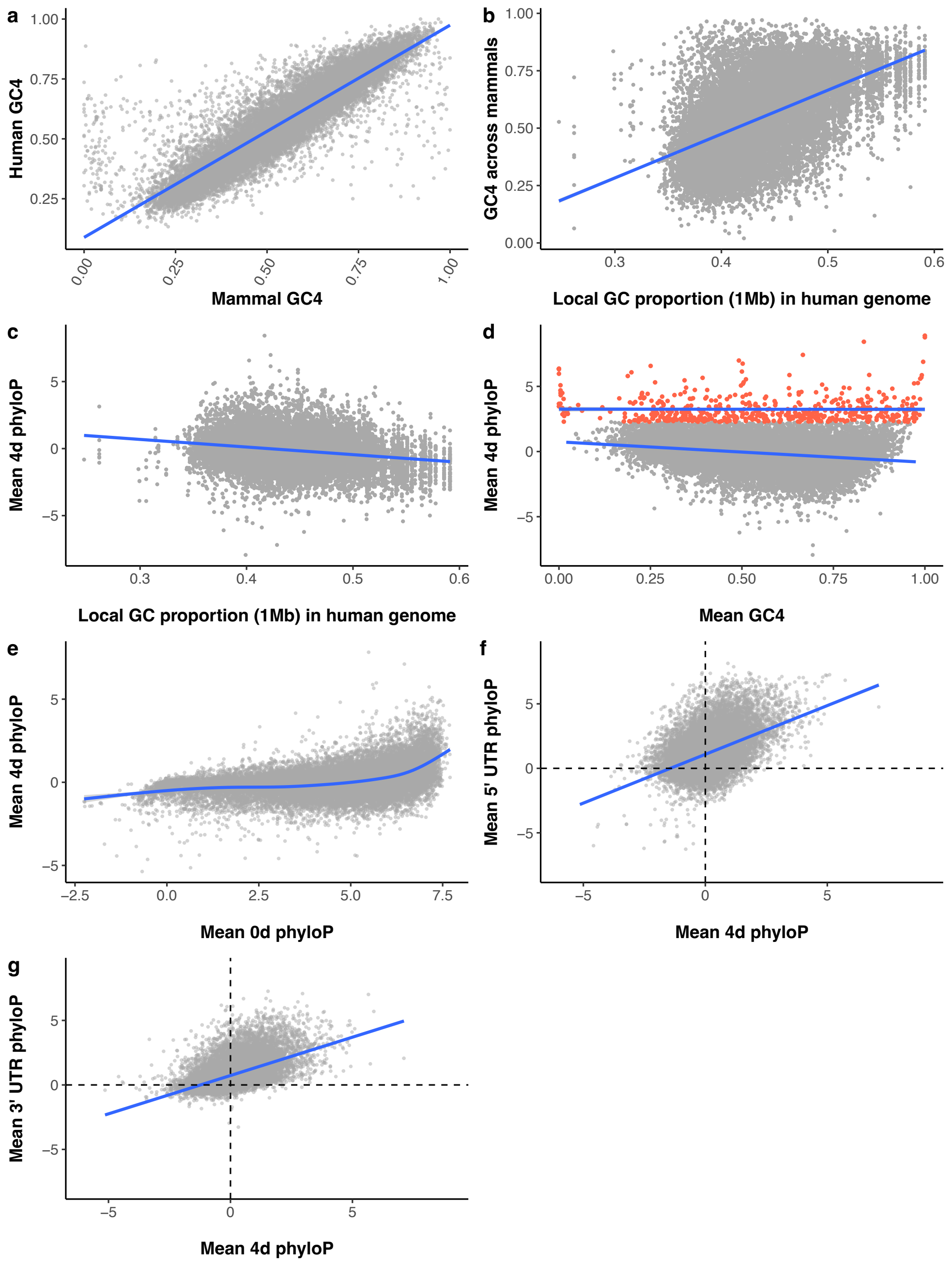

Figure S1. GC content and phyloP of four-fold degenerate sites. (a) GC4 content of human transcripts and summarized across the mammal genomes shows a strong positive correlation (n = 17,394 transcripts; Pearson's r = 0.90, $p < 2.2 \times 10^{-16}$). (b) Mammalian GC content at 4d sites per transcript significantly correlates with local GC content (1 Mb window) in the human genome, as expected under a model of GC-biased gene conversion (Pearson's r = 0.52, $p < 2.2 \times 10^{-16}$). (c) There is a negative correlation between local GC content and transcript 4d site mean phyloP (Pearson's r = -0.22, $p < 2.2 \times 10^{-16}$). (d) There is also a negative correlation between the mean GC4 content and mean 4d phyloP of transcripts (Pearson's r = -0.21, $p < 2.2 \times 10^{-16}$). For transcripts with a mean 4d phyloP >=

2.27, i.e. showing significant conservation (coloured red), the relationship is not significant (n = 532 transcripts; Pearson's r = -0.005, p = 0.91). Genes with the highest average 4d site phyloP are also the genes under the highest constraint generally, as evidenced by significant positive correlations between mean 4d phyloP and (e) mean 0d phyloP (Pearson's r = 0.42, $p < 2.2x10^{-16}$), (f) mean 5′ UTR phyloP (Pearson's r = 0.47, $p < 2.2x10^{-16}$), and (g) mean 3′ UTR phyloP (Pearson's r = 0.53, $p < 2.2x10^{-16}$). Blue lines show linear regressions in (a), (b), (c), (d), (f) and (g), and a general additive model in (e) using the 'geom_smooth' function in ggplot2 in R. Source data are provided as a Source Data file.

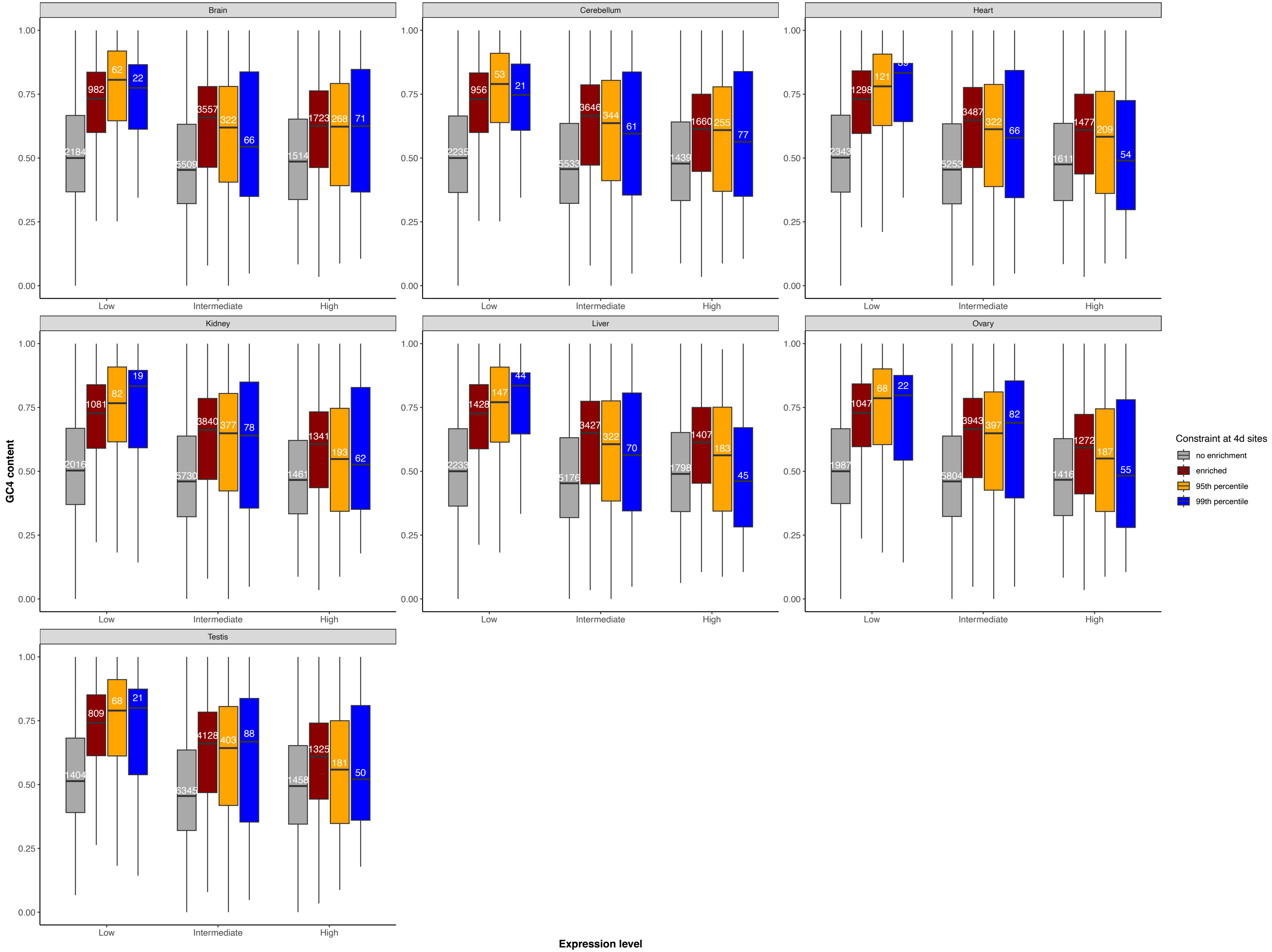
Brain
Cerebellum
Heart
Kidney
Liver
Ovary
Testis
GC4 content
Expression level
Low
Intermediate
High
Constraint at 4d sites
no enrichment
enriched
95th percentile
99th percentile

Figure S2. Highly expressed genes do not show higher GC content at 4d sites. Gene expression for each gene was classified as 'low' (bottom 20th percentile), 'intermediate'), and 'high' (top 20th percentile) and categorised by conserved 4d site enrichment. Overall and separately across seven distinct organs, GC4 content is 1) significantly higher in lowly compared to highly expressed genes (mean GC4 content of lowly and highly expressed genes = 0.59, and 0.54 respectively, ANOVA, df = 2, F = 440.9, $p < 2.2 \times 10^{-16}$) and 2) lowly expressed conserved 4d site-enriched genes have significantly higher GC4 content than highly expressed conserved 4d site-enriched genes (mean GC4 content of lowly and highly expressed conserved 4d site-enriched genes = 0.70, and 0.59 respectively, ANOVA, df = 2, F = 829.3, $p < 2.2 \times 10^{-16}$). Boxes represent first and third quartiles with median line, whiskers extend ±1.5 x IQR. Numbers in boxes show the number of transcripts per category. These findings suggest that high expression and related transcription-coupled repair cannot explain the high GC4 content observed at conserved 4d sites. Source data are provided as a Source Data file.

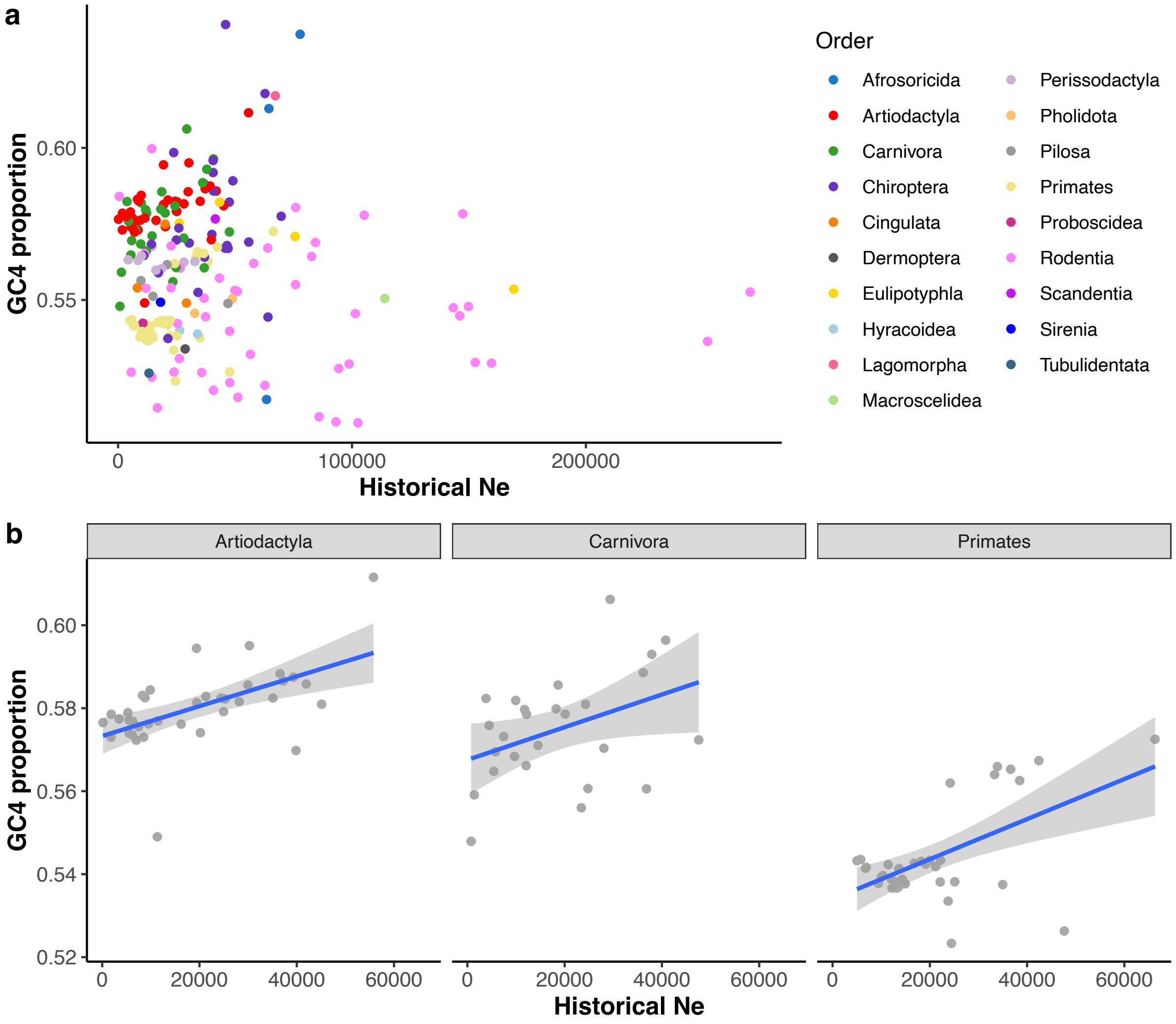

Figure S3. Correlations between GC content at 4d sites and historical effective population sizes in mammals. (a) Across all species, we do not observe a significant correlation between Historical N_e and GC4 proportion (n = 203, Pearson's r = -0.13, p = 0.073). (b) When considering within Order variation, there are significant positive correlations within Artiodactyla (n = 37, Pearson's r = 0.56, p = 3.1×10^{-4}), Carnivora (n = 26, Pearson's R = 0.40, p = 0.044), and Primates (n = 36, Pearson's r = 0.55, p = 4.5×10^{-4}). Blue lines and grey shading show linear regressions with 95% confidence using the 'geom_smooth' function in ggplot2 in R. Source data are provided as a Source Data file.

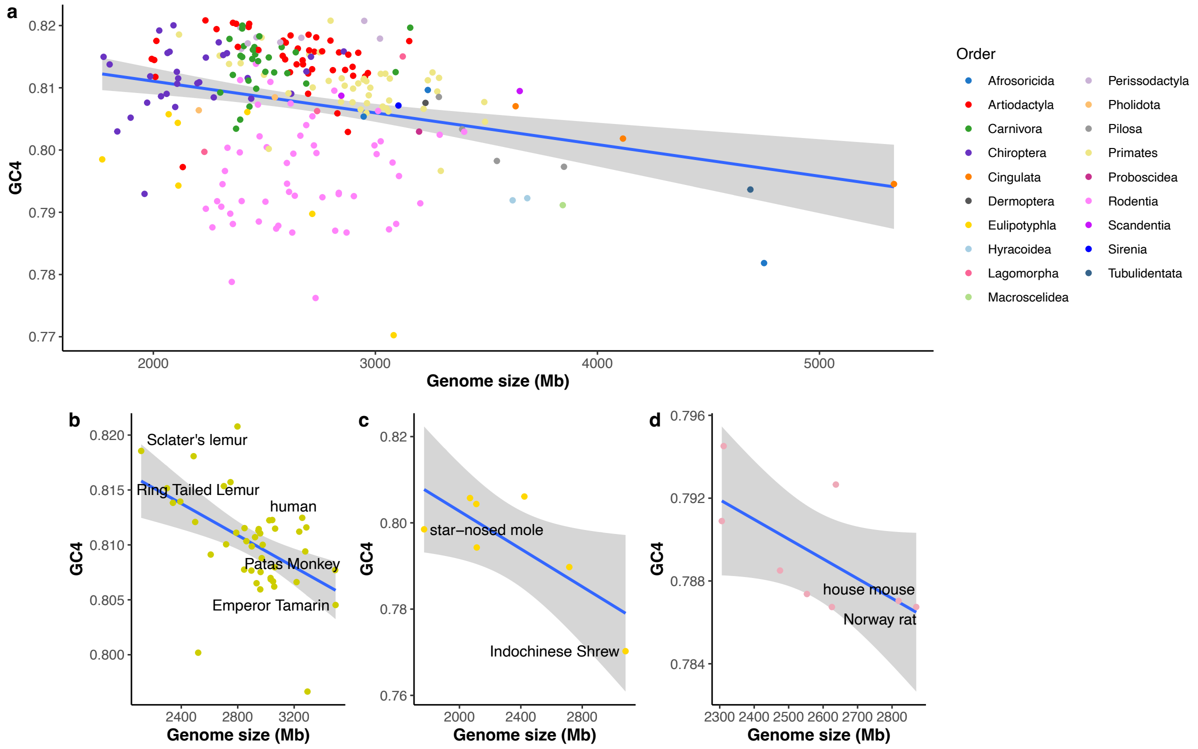

Figure S4. GC4 content at 4d sites present in all 240 genomes relates to genome size. There are significant negative correlations between GC4 content and genome size (a) across all 240 mammal genomes (Pearson's $r = -0.25$, $p = 8.9\times10^{-5}$), as well as within certain orders, including (b) Primates ($n = 43$; Pearson's $r = -0.50$, $p = 6.8\times10^{-4}$) and (c) Eulipotyphla ($n = 7$; Pearson's $r = -0.77$, $p = 0.044$). Within the Rodentia family Muridae there is a negative trend, but this is not significant with only 8 samples and little variation in genome size (Pearson's $r = -0.66$, $p = 0.075$). Blue lines and grey shading show linear regressions with 95% confidence intervals using the 'geom_smooth' function in ggplot2 in R. Source data are provided as a Source Data file.

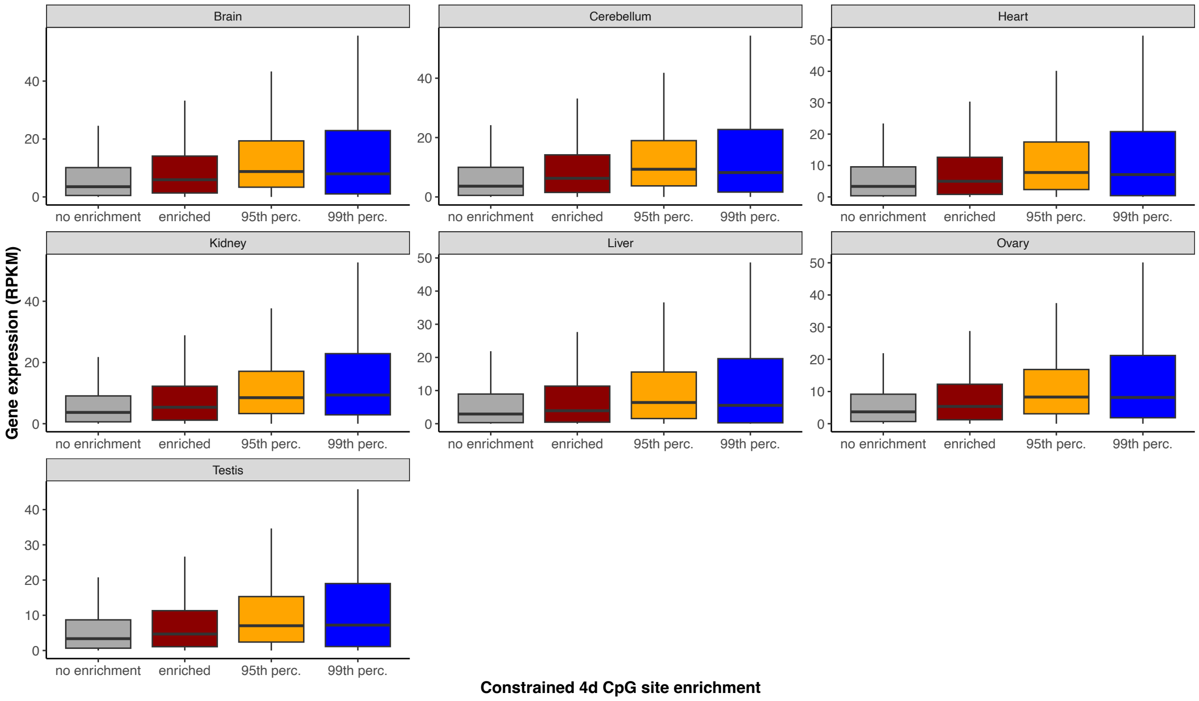

Figure S5. Conserved CpG enrichment relates to expression levels. Genes enriched for conserved 4d sites in CpGs (n = 3,925) have a higher mean expression than unenriched genes (n = 7,326); mean RPKM expression across all organs = 11.6 and 24.3, sd = 258 and 81.5 for genes lacking CpG enrichment and genes in the 99th percentile of conserved CpG enrichment respectively; ANOVA, df = 3, F = 24.44, $p = 8.28 \times 10^{-16}$; Tukey's HSD $p = 2.45 \times 10^{-12}$). This is predicted under the unwanted transcript hypothesis, where intragene methylation at CpG sites can prevent spurious transcription of highly active genes in regions of open chromatin. Boxes represent first and third quartiles with median line, whiskers extend ±1.5 x IQR. Source data are provided as a Source Data file.

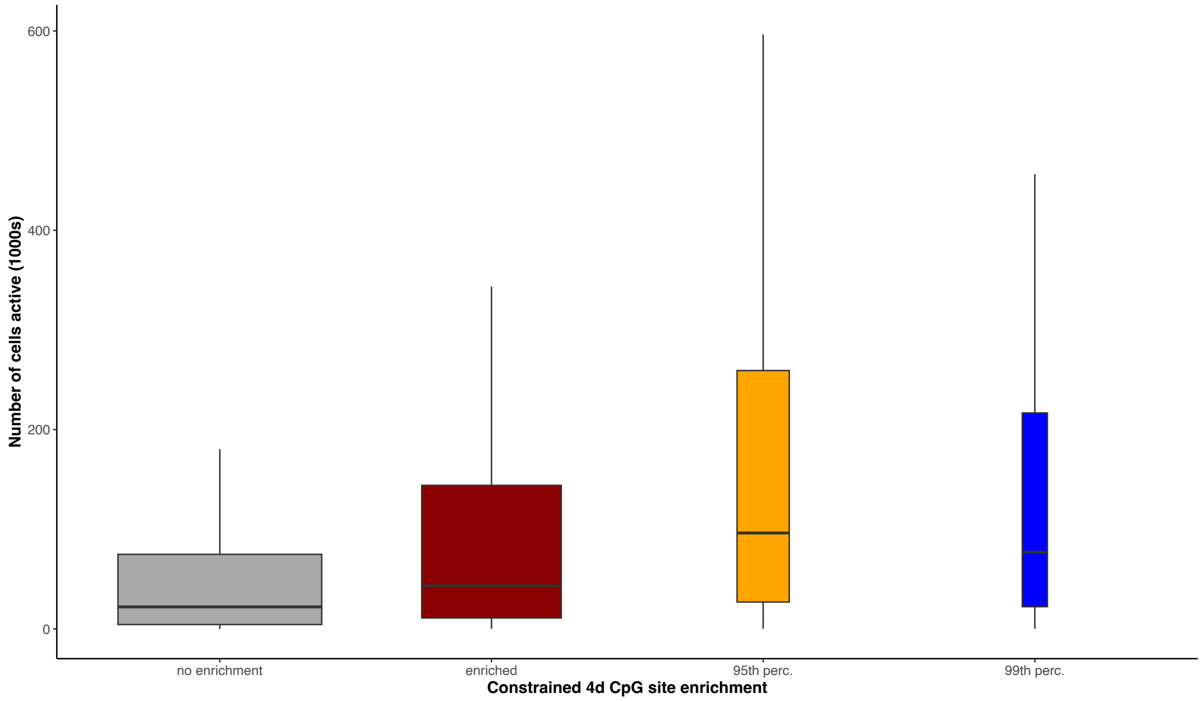

Figure S6. Conserved CpG enrichment relates to expression breadth. Genes with higher enrichment for conserved 4d sites in CpGs (95th percentile, n = 567 transcripts) are expressed in more cells in the mouse organogenesis cell atlas than those with lower enrichment (mean cell counts = 69,915 and 178,020 for genes lacking CpG enrichment and conserved CpG enriched genes respectively; ANOVA, df = 2, F = 195.9, p = $<2.2\times10^{-16}$; Tukey's HSD p = 7.65×10^{-9}). As in figure S5, this is predicted under the unwanted transcript hypothesis, where intragene methylation at CpG sites can prevent spurious transcription of highly active genes in regions of open chromatin. Boxes represent first and third quartiles with median line, whiskers extend ±1.5 x IQR. Box widths are relative to number of genes in each category. Source data are provided as a Source Data file.

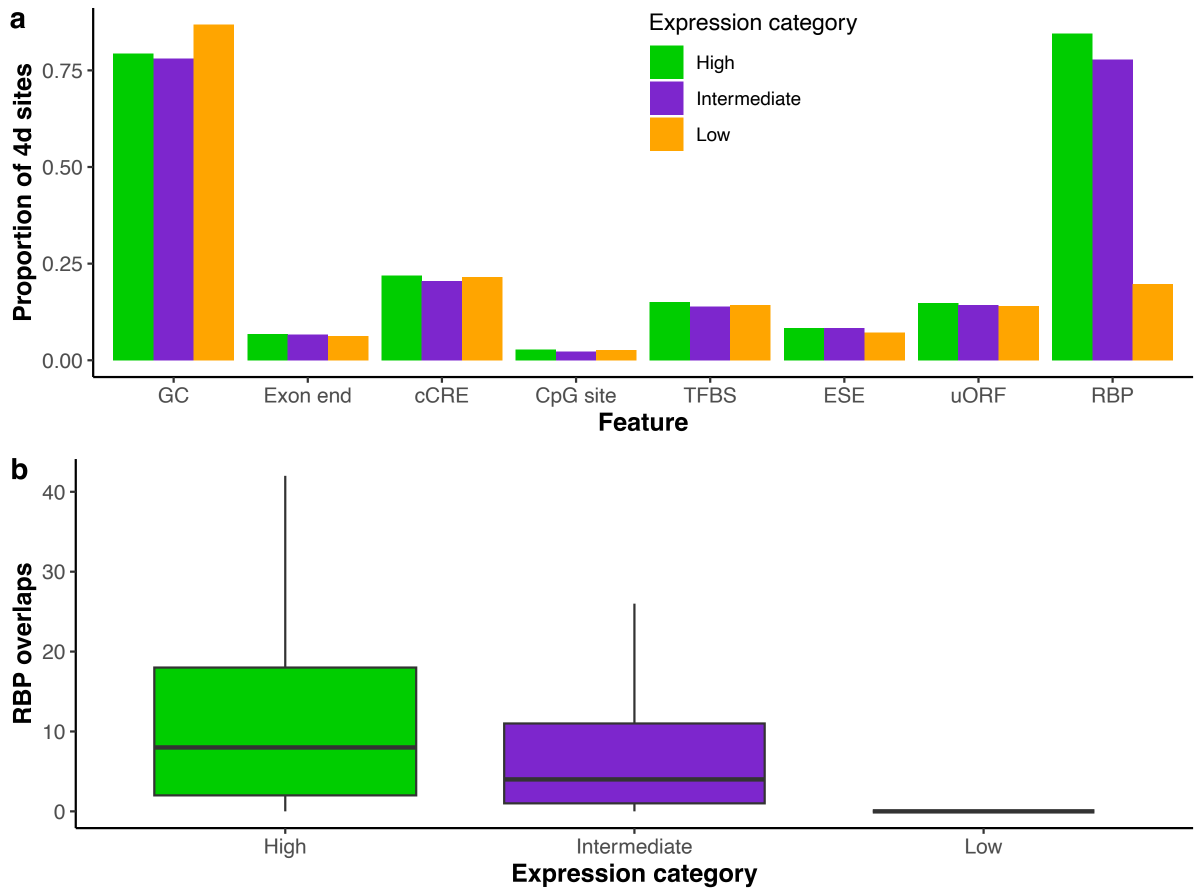

Figure S7. Little evidence that selection pressures acting on conserved 4d sites relate to gene expression levels. (a) Bar plot showing the proportions of conserved 4d sites (phyloP >= 2.27; n = 544,503) overlapping with potential secondary functions, as well as GC4 proportion, categorised by the expression level of the gene the 4d site is in. A general linear model comparing gene expression levels to functional feature overlap had an r^2 of 0.038, showing that very little of the variation in gene expression is explained by differences in overlap with these features. (b) The feature explaining most of the variance, with a relative importance of 97.5%, was overlap with RNA binding protein binding sites, where conserved 4d sites in genes with low expression show very little overlap with this feature (n = 5,687 high expression, 6,329 intermediate expression, and 5,306 low expression transcripts). Boxes represent first and third quartiles with median line, whiskers extend ±1.5 x IQR, outliers beyond whiskers are shown as points. Source data are provided as a Source Data file.
