# Supplementary material for: Interpreting mammalian synonymous site conservation in light of the unwanted transcript hypothesis
Source: Nat Commun. 2025 Feb 27;16:2007. doi: 10.1038/s41467-025-57179-w (PMC11865589; doi:10.1038/s41467-025-57179-w)
Supplement: Supplementary file 4 — Description of Additional Supplementary Files [file 41467_2025_57179_MOESM4_ESM.pdf]

Description of Additional Supplementary Files

File Name: Supplementary Data 1
Description: Locations in the human genome version GRCh38 of the 2,621,118 four-fold degenerate sites analysed in this study, in BED format.

File Name: Supplementary Data 2
Description: Summary statistics for 16,602 transcripts containing four-fold degenerate sites across mammals.

File Name: Supplementary Data 3
Description: Gene ontology enrichment results for genes enriched with conserved four-fold degenerate sites. Fisher's exact test with false discovery rate correction for multiple testing.

File Name: Supplementary Data 4
Description: Gene ontology enrichment results for genes enriched with conserved four-fold degenerate sites in CpG sites. Fisher's exact test with false discovery rate correction for multiple testing.

File Name: Supplementary Data 5
Description: Summary statistics of genes enriched for Polycomb Group transcription factor binding sites at conserved four-fold degenerate sites.

File Name: Supplementary Data 6
Description: Gene ontology enrichment results for genes enriched for Polycomb Group transcription factor binding sites at conserved four-fold degenerate sites. Fisher's exact test with false discovery rate correction for multiple testing.

File Name: Supplementary Data 7
Description: Summary statistics of genes absent of Polycomb Group transcription factor binding sites at conserved four-fold degenerate sites.

File Name: Supplementary Data 8
Description: Gene ontology enrichment results for genes absent of Polycomb Group transcription factor binding sites at conserved four-fold degenerate sites. Fisher's exact test with false discovery rate correction for multiple testing.

File Name: Supplementary Data 9
Description: Results from a general linear model of gene expression against overlap of four-fold degenerate sites with genomic features.
